# *catena*-Poly[[[aqua­(1,10-phenan­throline)cadmium(II)]-μ-benzene-1,4-dicarboxyl­ato] benzene-1,4-dicarboxylic acid hemisolvate]

**DOI:** 10.1107/S1600536809000889

**Published:** 2009-01-14

**Authors:** Zhuanzhuan Wang, Weihe Han, Zhihong Liu

**Affiliations:** a School of Chemistry and Materials Science, Shaanxi Normal University, Xi’an 710062, People’s Republic of China

## Abstract

A new cadmium(II) coordination polymer, {[Cd(C_8_H_4_O_4_)(C_12_H_8_N_2_)(H_2_O)]·0.5C_8_H_6_O_4_}_*n*_, has been synthesized under hydro­thermal conditions. The asymmetric unit contains one Cd^II^ atom, one benzene-1,4-dicarboxyl­ate anion, one 1,10-phenanthroline ligand, one coordinated water mol­ecule and half of an uncoordinated benzene-1,4-dicaboxylic acid solvent mol­ecule. The Cd^II^ atom is in the centre of a monocapped distorted octa­hedron made up of four O atoms of two chelating benzene-1,4-dicarboxyl­ate anions, one water O atom and two 1,10-phenanthroline N atoms. The metal centres are connected *via* bis-chelating benzene-1,4-dicarboxyl­ate anions into a zigzag chain structure along [001]. These chains are further connected by O—H⋯O hydrogen bonds between the water mol­ecules and adjacent carboxyl­ate O atoms. Additional O—H⋯O hydrogen bonding between the uncoordinated benzene-1,4-dicaboxylic acid mol­ecules along [010] consolidates the structure.

## Related literature

For background to coordination polymers, see: Liang *et al.* (2002 ▶); McGarrah *et al.* (2001 ▶); Moulton *et al.* (2002 ▶); Wu *et al.* (2007 ▶). Zheng *et al.* (2004 ▶). For related structures, see: Shi *et al.* (2004 ▶); Wang *et al.* (2004 ▶).
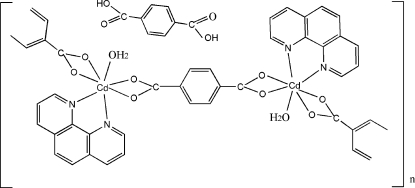


## Experimental

### 

#### Crystal data

[Cd(C_8_H_4_O_4_)(C_12_H_8_N_2_)(H_2_O)]·0.5C_8_H_6_O_4_
*M* *_r_* = 557.80
Monoclinic, 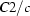

*a* = 26.108 (2) Å
*b* = 9.6928 (10) Å
*c* = 21.161 (2) Å
β = 126.494 (2)°
*V* = 4304.9 (7) Å^3^
*Z* = 8
Mo *K*α radiation
μ = 1.07 mm^−1^
*T* = 298 (2) K
0.42 × 0.18 × 0.02 mm

#### Data collection

Bruker SMART CCD diffractometer
Absorption correction: multi-scan (*SADABS*; Sheldrick, 2004 ▶) *T*_min_ = 0.663, *T*_max_ = 0.984
10895 measured reflections
3793 independent reflections
3061 reflections with *I* > 2σ(*I*)
*R*_int_ = 0.021

#### Refinement

*R*[*F* ^2^ > 2σ(*F* ^2^)] = 0.025
*wR*(*F* ^2^) = 0.062
*S* = 1.07
3793 reflections
309 parameters
H-atom parameters constrained
Δρ_max_ = 0.57 e Å^−3^
Δρ_min_ = −0.28 e Å^−3^

### 

Data collection: *SMART* (Bruker, 2001 ▶); cell refinement: *SAINT* (Bruker, 2001 ▶); data reduction: *SAINT*; program(s) used to solve structure: *SHELXS97* (Sheldrick, 2008 ▶); program(s) used to refine structure: *SHELXL97* (Sheldrick, 2008 ▶); molecular graphics: *SHELXTL* (Sheldrick, 2008 ▶); software used to prepare material for publication: *SHELXTL*.

## Supplementary Material

Crystal structure: contains datablocks I, global. DOI: 10.1107/S1600536809000889/wm2211sup1.cif

Structure factors: contains datablocks I. DOI: 10.1107/S1600536809000889/wm2211Isup2.hkl

Additional supplementary materials:  crystallographic information; 3D view; checkCIF report

## Figures and Tables

**Table 1:** Selected bond lengths (Å)

Cd1—O1	2.284 (3)
Cd1—O3	2.357 (2)
Cd1—N2	2.358 (2)
Cd1—N1	2.362 (2)
Cd1—O7	2.375 (2)
Cd1—O4	2.377 (2)
Cd1—O2	2.601 (3)

**Table 2:** Hydrogen-bond geometry (Å, °)

*D*—H⋯*A*	*D*—H	H⋯*A*	*D*⋯*A*	*D*—H⋯*A*
O5—H5⋯O6^i^	0.82	1.92	2.728 (3)	167
O7—H7*A*⋯O2^ii^	0.85	1.88	2.665 (3)	154
O7—H7*B*⋯O4^ii^	0.85	2.28	3.019 (3)	146

Symmetry codes: (i) 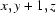; (ii) 
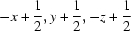
.

**Fig. 1.**
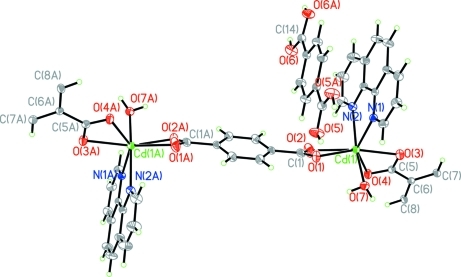
The asymmetric unit in the structure of compound (I). Displacement ellipsoids are drawn at the 30% probability level.

**Fig. 2.**
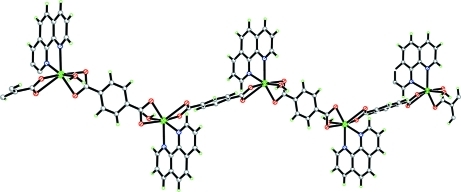
A view of a one-dimensional zigzag chain in the structure of compound (I). The uncoordinated 1,4-benzenedecaboxylic acid solvent molecules were omitted for clarity.

**Fig. 3.**
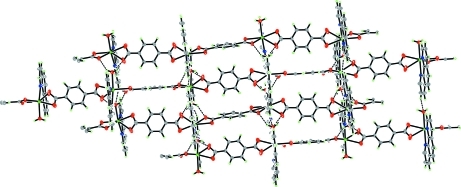
The layered structure of compound (I), constructed by H-bonding.

**Fig. 4.**
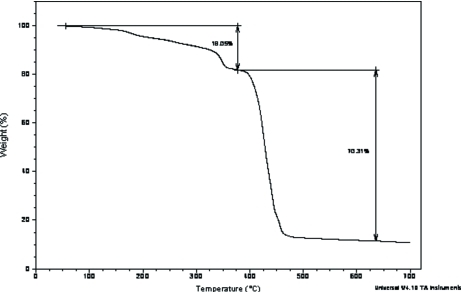
The TG-curve of the thermal decomposition of the title compound (I).

**Fig. 5.**
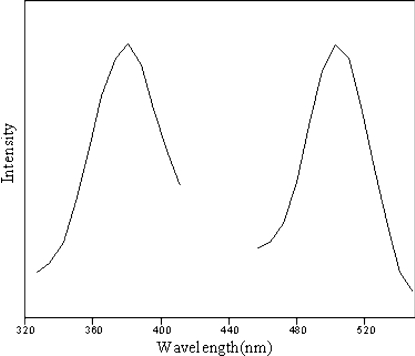
Solid-state emission spectra of the title complex (I) at room temperature.

## supplementary crystallographic information

### Comment

Carboxylic acid coordination polymers have an important position in coordination chemistry due to their interesting topologies and their potential applications in functional materials (Wu *et al.*, 2007). The coordination ability of the 1,10-phenanthroline ligand stems from its chelate effect which frequently results in the formation of low-dimensional coordination polymers because of its terminal-group effect (Moulton *et al.*, 2002). In this work, we report a new cadmium coordination polymer with a one-dimensional zigzag chain, {[Cd(C_8_H_4_O_4_)(C_12_H_8_N_2_)(H_2_O)]_2_(C_8_H_6_O_4_)}_n_, (I), that was synthesized from benzene-1,4-dicarboxylic acid, 1,10-phenanthroline and cadmium nitrate under hydrothermal conditions.

Each [Cd(C_8_H_4_O_4_)(C_12_H_8_N_2_)(H_2_O)]_2_(C_8_H_6_O_4_)] unit is composed of two Cd^2+^ ions, two 1,10-phenanthroline ligands, two benzene-1,4-dicarboxylate anions, two coordinated water molecules, and one uncoordinated neutral benzene-1,4-dicaboxylic acid solvent molecule. The Cd^2+^ ion in the complex is seven-coordinated with four oxygen atoms (O1, O2, O3 and O4) of two chelating bidentate carboxyl groups from two deprotonated benzene-1,4-dicaboxylic acid ligands, one O atom (O7) of a water molecule, and two nitrogen atoms (N1 and N2) from the chelating 1,10-phenanthroline ligand (Fig. 1). The Cd—O bond distances are between 2.284 (2) Å and 2.601 (2) Å, while the Cd—N bond distances are 2.358 (2) Å and 2.362 (2) Å, which all are in agreement with those of other reported Cd—O and Cd—N bond lengths (Wang *et al.*, 2004; Shi *et al.*, 2004). One Cd^2+^ ion connects two benzene-1,4-dicarboxylate ligands, and one benzene-1,4-dicarboxylic acid ligand also connects two Cd^2+^ ions, leading to a zigzag chain along [001], as shown in Fig. 2. These chains are further connected through O—H···O hydrogen bonding interactions between the water molecules and the oxygen atoms of the coordinated carboxyl groups of benzene-1,4-dicarboxylate ligands in neighboring chains to give an extended supramolecular two-dimensional layer structure, as illustrated in Fig. 3. Additional O—H···O hydrogen bonding between the uncoordinated benzene-1,4-dicaboxylic acid molecules along [010] consolidates the structure.

### Experimental

All regents used in the synthesis were of analytic grade and were used without further purification. A mixture of Cd(NO_3_)_2_^.^6H_2_O (0.3085 g), 1,10-phenanthroline (0.099 g), benzene-1,4-dicarboxylic acid (0.083 g) and water (20 mL) was sealed in a 40 mL stainless steel reactor with a Teflon inlay after adjustment of the pH to 7 by addition of a dilute NaOH solution. The autoclave was heated to 448 K for 6 d, and then cooled to room temperature. Colorless hexagonal-prismatic crystals were obtained. Elemental analysis, calculated: C, 51.68, H, 3.07, N 5.02; found: C, 50.87, H, 3.22, N 4.96.

The title compoud was further characterized by FT-IR spectroscopy (recorded over the 400 to 4000 cm^-1^ region on a Nicolet NEXUS 670 spectrometer with KBr pellets at room temperature); by thermogravimetric analysis (TGA) (performed on a Universal V4.1D TA-SDT Q600 thermal analyzer in N_2_ atmosphere with a heating rate of 10 °/min) and by its luminescent properties of the solid state under room temperature.

The FT-IR spectrum of the title compound exhibited the following absorption bands that were assigned referring to literature (Liang *et al.*, 2002). A broad band at 3199 cm^-1^ corresponds to the O-H stretching vibration of the coordinated water molecule. The peak at 1682 cm^-1^ is attributed to the C=O stretching vibration of the carboxylate group, and the peak at 1380 cm^-1^ is due to the C-O stretching vibration of carboxylate group. A typical TG curve is shown in Figure 4. It shows that this compound has two steps of mass loss between 55 and 635 °C. The first step is completed at 376 °C, accompanied with 18.05 % mass loss, which is in good agreement with the theoretical mass loss of 18.12 %, corresponding to the evaporation of two water molecules and an uncoordinated neutral 1,4-benzenedecaboxylic acid molecule. In the second step, the mass loss is 70.31 % from 376 to 635 °C, which corresponds to the decomposition of two 1,10-phenanthroline and two deprotonated 1,4-benzenedecaboxylic acid ligands and can be compared with the calculated value of 70.37 %. After the two steps of mass loss, the mass fraction of the residue is 11.44 %, which is in agreement with the calculated mass summation of the remaining CdO (11.51 %). Previous studies have shown that coordination polymers containing zinc and cadmium ions exhibit photoluminescent properties (McGarrah *et al.*, 2001). As shown in Figure 5, upon excitation of the solid sample at 370 nm, it exhibited strong cyan fluorescent emission bands at 504 nm. It is probably due to the (π^*^-π) transitions changing into the (π^*^-n) transitions after forming the coordination polymer (Zheng *et al.*, 2004).

### Refinement

H atoms were positioned in calculated positions (O–H = 0.82 Å and 0.82 Å, C–H = 0.93 Å) and were refined using the riding model approximation with U_iso_(H) = 1.2U_eq_ of the parent atom.

### Crystal data

**Table:**

[Cd(C_8_H_4_O_4_)(C_12_H_8_N_2_)(H_2_O)]·0.5C_8_H_6_O_4_	*F*(000) = 2232
*M**_r_* = 557.80	*D*_x_ = 1.721 Mg m^−^^3^
Monoclinic, *C*2/*c*	Mo *K*α radiation, λ = 0.71073 Å
Hall symbol: -C 2yc	Cell parameters from 5356 reflections
*a* = 26.108 (2) Å	θ = 2.4–27.4°
*b* = 9.6928 (10) Å	µ = 1.07 mm^−^^1^
*c* = 21.161 (2) Å	*T* = 298 K
β = 126.494 (2)°	Prism, colourless
*V* = 4304.9 (7) Å^3^	0.42 × 0.18 × 0.02 mm
*Z* = 8	

### Data collection

**Table:**

Bruker SMART CCD diffractometer	3793 independent reflections
Radiation source: fine-focus sealed tube	3061 reflections with *I* > 2σ(*I*)
graphite	*R*_int_ = 0.021
φ and ω scans	θ_max_ = 25.0°, θ_min_ = 2.0°
Absorption correction: multi-scan (*SADABS*; Sheldrick, 2004)	*h* = −30→30
*T*_min_ = 0.663, *T*_max_ = 0.984	*k* = −11→10
10895 measured reflections	*l* = −18→25

### Refinement

**Table:**

Refinement on *F*^2^	Primary atom site location: structure-invariant direct methods
Least-squares matrix: full	Secondary atom site location: difference Fourier map
*R*[*F*^2^ > 2σ(*F*^2^)] = 0.025	Hydrogen site location: inferred from neighbouring sites
*wR*(*F*^2^) = 0.062	H-atom parameters constrained
*S* = 1.07	*w* = 1/[σ^2^(*F*_o_^2^) + (0.0233*P*)^2^ + 5.6765*P*] where *P* = (*F*_o_^2^ + 2*F*_c_^2^)/3
3793 reflections	(Δ/σ)_max_ = 0.001
309 parameters	Δρ_max_ = 0.57 e Å^−^^3^
0 restraints	Δρ_min_ = −0.28 e Å^−^^3^

### Special details

**Table:**

Geometry. All e.s.d.'s (except the e.s.d. in the dihedral angle between two l.s. planes) are estimated using the full covariance matrix. The cell e.s.d.'s are taken into account individually in the estimation of e.s.d.'s in distances, angles and torsion angles; correlations between e.s.d.'s in cell parameters are only used when they are defined by crystal symmetry. An approximate (isotropic) treatment of cell e.s.d.'s is used for estimating e.s.d.'s involving l.s. planes.
Refinement. Refinement of *F*^2^ against ALL reflections. The weighted *R*-factor *wR* and goodness of fit *S* are based on *F*^2^, conventional *R*-factors *R* are based on *F*, with *F* set to zero for negative *F*^2^. The threshold expression of *F*^2^ > σ(*F*^2^) is used only for calculating *R*-factors(gt) *etc*. and is not relevant to the choice of reflections for refinement. *R*-factors based on *F*^2^ are statistically about twice as large as those based on *F*, and *R*- factors based on ALL data will be even larger.

### Fractional atomic coordinates and isotropic or equivalent isotropic displacement parameters (Å^2^)

**Table:**

	*x*	*y*	*z*	*U*_iso_*/*U*_eq_	Occ. (<1)
Cd1	0.163966 (9)	0.80224 (2)	0.186508 (12)	0.03638 (8)	
N1	0.05395 (11)	0.8470 (2)	0.11727 (14)	0.0355 (5)	
N2	0.10891 (11)	0.5924 (2)	0.15983 (14)	0.0415 (6)	
O1	0.18828 (13)	0.8727 (3)	0.30445 (14)	0.0762 (8)	
O2	0.23664 (13)	0.6773 (4)	0.32126 (16)	0.0928 (10)	
O3	0.14899 (10)	0.7998 (2)	0.06513 (12)	0.0509 (6)	
O4	0.24360 (10)	0.7435 (2)	0.16977 (12)	0.0502 (6)	
O5	0.05219 (11)	1.1134 (2)	0.28343 (16)	0.0729 (8)	
H5	0.0460	1.1969	0.2794	0.109*	0.50
O6	0.05277 (11)	0.3946 (2)	0.28845 (15)	0.0701 (7)	
H6	0.0467	0.3111	0.2840	0.105*	0.50
O7	0.18243 (10)	1.0437 (2)	0.19469 (12)	0.0504 (6)	
H7A	0.2076	1.0625	0.1829	0.061*	
H7B	0.1988	1.0725	0.2410	0.061*	
C1	0.22048 (16)	0.7708 (4)	0.3456 (2)	0.0569 (10)	
C2	0.23658 (13)	0.7594 (3)	0.42642 (17)	0.0382 (7)	
C3	0.23231 (15)	0.8742 (3)	0.46128 (19)	0.0478 (8)	
H3	0.2202	0.9585	0.4351	0.057*	
C4	0.25404 (15)	0.6350 (3)	0.46504 (19)	0.0477 (8)	
H4	0.2567	0.5570	0.4415	0.057*	
C5	0.20622 (14)	0.7676 (3)	0.09733 (18)	0.0373 (7)	
C6	0.22902 (13)	0.7568 (3)	0.04736 (17)	0.0332 (6)	
C7	0.18645 (13)	0.7628 (3)	−0.03373 (18)	0.0425 (7)	
H7	0.1432	0.7718	−0.0571	0.051*	
C8	0.29305 (14)	0.7441 (3)	0.08069 (18)	0.0428 (7)	
H8	0.3226	0.7403	0.1351	0.051*	
C9	0.0000	1.0513 (4)	0.2500	0.0443 (11)	
C10	0.0000	0.8973 (4)	0.2500	0.0390 (10)	
C11	0.05674 (14)	0.8252 (3)	0.28751 (19)	0.0479 (8)	
H11	0.0951	0.8730	0.3127	0.058*	
C12	0.05684 (15)	0.6829 (3)	0.2878 (2)	0.0500 (8)	
H12	0.0952	0.6352	0.3134	0.060*	
C13	0.0000	0.6107 (4)	0.2500	0.0400 (10)	
C14	0.0000	0.4565 (5)	0.2500	0.0452 (11)	
C15	0.02751 (15)	0.9707 (3)	0.09881 (19)	0.0470 (8)	
H15	0.0539	1.0478	0.1175	0.056*	
C16	−0.03804 (15)	0.9903 (4)	0.0527 (2)	0.0538 (9)	
H16	−0.0549	1.0790	0.0409	0.065*	
C17	−0.07692 (15)	0.8796 (4)	0.0251 (2)	0.0547 (9)	
H17	−0.1209	0.8918	−0.0067	0.066*	
C18	−0.05130 (14)	0.7468 (3)	0.04402 (19)	0.0452 (8)	
C19	0.01571 (13)	0.7355 (3)	0.09130 (16)	0.0344 (6)	
C20	0.04452 (14)	0.6005 (3)	0.11358 (17)	0.0390 (7)	
C21	0.00562 (16)	0.4833 (3)	0.0881 (2)	0.0520 (8)	
C22	0.0362 (2)	0.3557 (4)	0.1137 (3)	0.0774 (12)	
H22	0.0120	0.2754	0.0981	0.093*	
C23	0.1001 (2)	0.3473 (4)	0.1607 (3)	0.0796 (13)	
H23	0.1204	0.2622	0.1781	0.096*	
C24	0.13532 (18)	0.4689 (4)	0.1827 (2)	0.0626 (10)	
H24	0.1795	0.4628	0.2152	0.075*	
C25	−0.08894 (16)	0.6240 (4)	0.0181 (2)	0.0647 (10)	
H25	−0.1331	0.6313	−0.0142	0.078*	
C26	−0.06213 (18)	0.4987 (4)	0.0391 (2)	0.0692 (11)	
H26	−0.0879	0.4207	0.0217	0.083*	

### Atomic displacement parameters (Å^2^)

**Table:**

	*U*^11^	*U*^22^	*U*^33^	*U*^12^	*U*^13^	*U*^23^
Cd1	0.03152 (12)	0.04161 (13)	0.03571 (13)	0.00181 (10)	0.01983 (10)	0.00116 (11)
N1	0.0354 (13)	0.0350 (13)	0.0368 (14)	0.0012 (11)	0.0219 (12)	0.0022 (11)
N2	0.0383 (14)	0.0368 (14)	0.0448 (15)	0.0046 (11)	0.0223 (13)	−0.0005 (12)
O1	0.085 (2)	0.091 (2)	0.0430 (15)	−0.0133 (17)	0.0329 (15)	0.0057 (15)
O2	0.0747 (19)	0.157 (3)	0.0568 (17)	0.0122 (19)	0.0443 (16)	−0.0235 (19)
O3	0.0394 (12)	0.0742 (15)	0.0446 (12)	0.0144 (11)	0.0280 (11)	0.0073 (12)
O4	0.0433 (12)	0.0731 (15)	0.0366 (13)	0.0063 (11)	0.0251 (11)	0.0046 (11)
O5	0.0487 (14)	0.0436 (15)	0.101 (2)	−0.0060 (12)	0.0307 (15)	−0.0069 (14)
O6	0.0583 (16)	0.0435 (14)	0.0852 (19)	0.0088 (12)	0.0300 (15)	−0.0008 (14)
O7	0.0564 (13)	0.0473 (13)	0.0485 (13)	−0.0114 (11)	0.0317 (12)	−0.0060 (11)
C1	0.0359 (18)	0.091 (3)	0.044 (2)	−0.0160 (19)	0.0237 (17)	−0.012 (2)
C2	0.0297 (15)	0.0521 (18)	0.0326 (16)	−0.0034 (13)	0.0185 (14)	−0.0059 (15)
C3	0.0518 (19)	0.0381 (18)	0.0470 (19)	0.0049 (15)	0.0260 (17)	0.0044 (15)
C4	0.0513 (19)	0.0443 (19)	0.049 (2)	0.0033 (15)	0.0302 (17)	−0.0118 (17)
C5	0.0387 (17)	0.0345 (16)	0.0414 (18)	0.0001 (13)	0.0253 (15)	0.0002 (13)
C6	0.0339 (15)	0.0303 (14)	0.0367 (16)	−0.0003 (12)	0.0217 (14)	−0.0008 (13)
C7	0.0277 (15)	0.059 (2)	0.0396 (18)	0.0030 (14)	0.0196 (14)	0.0025 (15)
C8	0.0337 (16)	0.0604 (19)	0.0299 (16)	0.0016 (14)	0.0166 (14)	−0.0007 (15)
C9	0.046 (3)	0.042 (3)	0.040 (3)	0.000	0.023 (2)	0.000
C10	0.042 (2)	0.040 (2)	0.034 (2)	0.000	0.023 (2)	0.000
C11	0.0376 (17)	0.044 (2)	0.055 (2)	−0.0039 (14)	0.0236 (16)	−0.0058 (16)
C12	0.0386 (18)	0.046 (2)	0.057 (2)	0.0050 (15)	0.0235 (17)	0.0006 (16)
C13	0.043 (2)	0.039 (3)	0.038 (2)	0.000	0.024 (2)	0.000
C14	0.048 (3)	0.044 (3)	0.041 (3)	0.000	0.025 (2)	0.000
C15	0.0451 (19)	0.0393 (18)	0.058 (2)	0.0037 (14)	0.0311 (17)	0.0069 (16)
C16	0.0446 (19)	0.047 (2)	0.066 (2)	0.0145 (16)	0.0312 (19)	0.0146 (18)
C17	0.0318 (17)	0.072 (3)	0.053 (2)	0.0116 (17)	0.0216 (16)	0.0107 (19)
C18	0.0329 (16)	0.0537 (19)	0.0446 (19)	−0.0028 (14)	0.0207 (15)	−0.0013 (16)
C19	0.0344 (15)	0.0394 (17)	0.0301 (15)	−0.0020 (13)	0.0195 (13)	−0.0014 (13)
C20	0.0405 (17)	0.0398 (17)	0.0377 (17)	−0.0029 (13)	0.0238 (15)	−0.0045 (14)
C21	0.054 (2)	0.044 (2)	0.057 (2)	−0.0094 (16)	0.0331 (19)	−0.0105 (17)
C22	0.084 (3)	0.039 (2)	0.105 (4)	−0.011 (2)	0.054 (3)	−0.013 (2)
C23	0.085 (3)	0.034 (2)	0.110 (4)	0.007 (2)	0.052 (3)	0.001 (2)
C24	0.058 (2)	0.045 (2)	0.072 (3)	0.0138 (18)	0.031 (2)	0.0034 (19)
C25	0.0352 (19)	0.077 (3)	0.065 (2)	−0.0120 (19)	0.0211 (18)	−0.009 (2)
C26	0.056 (2)	0.062 (3)	0.082 (3)	−0.027 (2)	0.037 (2)	−0.020 (2)

### Geometric parameters (Å, °)

**Table:**

Cd1—O1	2.284 (3)	C8—C7^ii^	1.382 (4)
Cd1—O3	2.357 (2)	C8—H8	0.9300
Cd1—N2	2.358 (2)	C9—O5^iii^	1.254 (3)
Cd1—N1	2.362 (2)	C9—C10	1.493 (6)
Cd1—O7	2.375 (2)	C10—C11^iii^	1.384 (4)
Cd1—O4	2.377 (2)	C10—C11	1.384 (4)
Cd1—O2	2.601 (3)	C11—C12	1.379 (4)
N1—C15	1.321 (4)	C11—H11	0.9300
N1—C19	1.347 (4)	C12—C13	1.385 (4)
N2—C24	1.321 (4)	C12—H12	0.9300
N2—C20	1.354 (4)	C13—C12^iii^	1.385 (4)
O1—C1	1.253 (4)	C13—C14	1.494 (6)
O2—C1	1.235 (4)	C14—O6^iii^	1.260 (3)
O3—C5	1.258 (3)	C15—C16	1.390 (4)
O4—C5	1.256 (3)	C15—H15	0.9300
O5—C9	1.254 (3)	C16—C17	1.348 (5)
O5—H5	0.8200	C16—H16	0.9300
O6—C14	1.260 (3)	C17—C18	1.395 (5)
O6—H6	0.8200	C17—H17	0.9300
O7—H7A	0.8501	C18—C19	1.411 (4)
O7—H7B	0.8499	C18—C25	1.429 (5)
C1—C2	1.503 (5)	C19—C20	1.442 (4)
C2—C4	1.374 (4)	C20—C21	1.400 (4)
C2—C3	1.376 (4)	C21—C22	1.395 (5)
C3—C4^i^	1.380 (5)	C21—C26	1.430 (5)
C3—H3	0.9300	C22—C23	1.345 (5)
C4—C3^i^	1.380 (5)	C22—H22	0.9300
C4—H4	0.9300	C23—C24	1.393 (5)
C5—C6	1.494 (4)	C23—H23	0.9300
C6—C8	1.380 (4)	C24—H24	0.9300
C6—C7	1.384 (4)	C25—C26	1.339 (5)
C7—C8^ii^	1.382 (4)	C25—H25	0.9300
C7—H7	0.9300	C26—H26	0.9300
			
O1—Cd1—O3	162.20 (9)	C6—C8—C7^ii^	120.4 (3)
O1—Cd1—N2	104.69 (9)	C6—C8—H8	119.8
O3—Cd1—N2	92.75 (8)	C7^ii^—C8—H8	119.8
O1—Cd1—N1	93.90 (9)	O5—C9—O5^iii^	122.6 (4)
O3—Cd1—N1	88.48 (7)	O5—C9—C10	118.7 (2)
N2—Cd1—N1	70.53 (8)	O5^iii^—C9—C10	118.7 (2)
O1—Cd1—O7	73.32 (9)	C11^iii^—C10—C11	119.3 (4)
O3—Cd1—O7	89.10 (8)	C11^iii^—C10—C9	120.3 (2)
N2—Cd1—O7	159.42 (8)	C11—C10—C9	120.3 (2)
N1—Cd1—O7	89.04 (8)	C12—C11—C10	120.4 (3)
O1—Cd1—O4	122.37 (9)	C12—C11—H11	119.8
O3—Cd1—O4	55.12 (7)	C10—C11—H11	119.8
N2—Cd1—O4	102.73 (8)	C11—C12—C13	120.3 (3)
N1—Cd1—O4	143.17 (8)	C11—C12—H12	119.9
O7—Cd1—O4	95.13 (8)	C13—C12—H12	119.9
O1—Cd1—O2	52.68 (10)	C12—C13—C12^iii^	119.3 (4)
O3—Cd1—O2	136.90 (8)	C12—C13—C14	120.3 (2)
N2—Cd1—O2	78.69 (10)	C12^iii^—C13—C14	120.3 (2)
N1—Cd1—O2	126.21 (9)	O6^iii^—C14—O6	123.1 (4)
O7—Cd1—O2	113.27 (9)	O6^iii^—C14—C13	118.5 (2)
O4—Cd1—O2	85.37 (8)	O6—C14—C13	118.5 (2)
C15—N1—C19	118.5 (2)	N1—C15—C16	122.7 (3)
C15—N1—Cd1	125.38 (19)	N1—C15—H15	118.6
C19—N1—Cd1	115.99 (18)	C16—C15—H15	118.6
C24—N2—C20	118.1 (3)	C17—C16—C15	119.4 (3)
C24—N2—Cd1	125.7 (2)	C17—C16—H16	120.3
C20—N2—Cd1	116.18 (19)	C15—C16—H16	120.3
C1—O1—Cd1	99.2 (2)	C16—C17—C18	120.1 (3)
C1—O2—Cd1	84.7 (2)	C16—C17—H17	120.0
C5—O3—Cd1	92.19 (18)	C18—C17—H17	120.0
C5—O4—Cd1	91.35 (17)	C17—C18—C19	117.1 (3)
C9—O5—H5	109.5	C17—C18—C25	123.7 (3)
C14—O6—H6	109.5	C19—C18—C25	119.1 (3)
Cd1—O7—H7A	110.4	N1—C19—C18	122.2 (3)
Cd1—O7—H7B	110.4	N1—C19—C20	118.6 (2)
H7A—O7—H7B	108.7	C18—C19—C20	119.2 (3)
O2—C1—O1	122.9 (4)	N2—C20—C21	122.4 (3)
O2—C1—C2	119.2 (4)	N2—C20—C19	118.1 (3)
O1—C1—C2	117.8 (3)	C21—C20—C19	119.5 (3)
C4—C2—C3	119.7 (3)	C22—C21—C20	117.0 (3)
C4—C2—C1	120.7 (3)	C22—C21—C26	123.3 (3)
C3—C2—C1	119.6 (3)	C20—C21—C26	119.7 (3)
C2—C3—C4^i^	120.3 (3)	C23—C22—C21	120.8 (4)
C2—C3—H3	119.9	C23—C22—H22	119.6
C4^i^—C3—H3	119.9	C21—C22—H22	119.6
C2—C4—C3^i^	120.0 (3)	C22—C23—C24	118.6 (4)
C2—C4—H4	120.0	C22—C23—H23	120.7
C3^i^—C4—H4	120.0	C24—C23—H23	120.7
O4—C5—O3	121.2 (3)	N2—C24—C23	123.2 (3)
O4—C5—C6	120.2 (3)	N2—C24—H24	118.4
O3—C5—C6	118.6 (3)	C23—C24—H24	118.4
C8—C6—C7	118.2 (3)	C26—C25—C18	121.6 (3)
C8—C6—C5	121.1 (3)	C26—C25—H25	119.2
C7—C6—C5	120.7 (3)	C18—C25—H25	119.2
C8^ii^—C7—C6	121.4 (3)	C25—C26—C21	120.8 (3)
C8^ii^—C7—H7	119.3	C25—C26—H26	119.6
C6—C7—H7	119.3	C21—C26—H26	119.6
			
O1—Cd1—N1—C15	73.5 (3)	Cd1—O4—C5—C6	176.8 (2)
O3—Cd1—N1—C15	−88.8 (3)	Cd1—O3—C5—O4	4.1 (3)
N2—Cd1—N1—C15	177.7 (3)	Cd1—O3—C5—C6	−176.7 (2)
O7—Cd1—N1—C15	0.3 (3)	O4—C5—C6—C8	−11.4 (4)
O4—Cd1—N1—C15	−97.0 (3)	O3—C5—C6—C8	169.4 (3)
O2—Cd1—N1—C15	118.8 (2)	O4—C5—C6—C7	170.3 (3)
O1—Cd1—N1—C19	−110.4 (2)	O3—C5—C6—C7	−8.9 (4)
O3—Cd1—N1—C19	87.2 (2)	C8—C6—C7—C8^ii^	0.3 (5)
N2—Cd1—N1—C19	−6.22 (19)	C5—C6—C7—C8^ii^	178.6 (3)
O7—Cd1—N1—C19	176.4 (2)	C7—C6—C8—C7^ii^	−0.3 (5)
O4—Cd1—N1—C19	79.1 (2)	C5—C6—C8—C7^ii^	−178.6 (3)
O2—Cd1—N1—C19	−65.1 (2)	O5—C9—C10—C11^iii^	−178.8 (2)
O1—Cd1—N2—C24	−88.3 (3)	O5^iii^—C9—C10—C11^iii^	1.2 (2)
O3—Cd1—N2—C24	95.3 (3)	O5—C9—C10—C11	1.2 (2)
N1—Cd1—N2—C24	−177.3 (3)	O5^iii^—C9—C10—C11	−178.8 (2)
O7—Cd1—N2—C24	−169.9 (3)	C11^iii^—C10—C11—C12	−0.2 (2)
O4—Cd1—N2—C24	40.5 (3)	C9—C10—C11—C12	179.8 (2)
O2—Cd1—N2—C24	−42.1 (3)	C10—C11—C12—C13	0.4 (5)
O1—Cd1—N2—C20	95.2 (2)	C11—C12—C13—C12^iii^	−0.2 (2)
O3—Cd1—N2—C20	−81.1 (2)	C11—C12—C13—C14	179.8 (2)
N1—Cd1—N2—C20	6.3 (2)	C12—C13—C14—O6^iii^	−176.3 (2)
O7—Cd1—N2—C20	13.6 (4)	C12^iii^—C13—C14—O6^iii^	3.7 (2)
O4—Cd1—N2—C20	−136.0 (2)	C12—C13—C14—O6	3.7 (2)
O2—Cd1—N2—C20	141.5 (2)	C12^iii^—C13—C14—O6	−176.3 (2)
O3—Cd1—O1—C1	−133.0 (3)	C19—N1—C15—C16	−1.1 (5)
N2—Cd1—O1—C1	58.9 (2)	Cd1—N1—C15—C16	174.8 (2)
N1—Cd1—O1—C1	129.8 (2)	N1—C15—C16—C17	−0.1 (5)
O7—Cd1—O1—C1	−142.3 (2)	C15—C16—C17—C18	1.1 (5)
O4—Cd1—O1—C1	−56.9 (2)	C16—C17—C18—C19	−1.0 (5)
O2—Cd1—O1—C1	−4.0 (2)	C16—C17—C18—C25	179.3 (3)
O1—Cd1—O2—C1	4.0 (2)	C15—N1—C19—C18	1.3 (4)
O3—Cd1—O2—C1	163.68 (19)	Cd1—N1—C19—C18	−175.1 (2)
N2—Cd1—O2—C1	−114.5 (2)	C15—N1—C19—C20	−177.9 (3)
N1—Cd1—O2—C1	−59.1 (3)	Cd1—N1—C19—C20	5.7 (3)
O7—Cd1—O2—C1	47.9 (2)	C17—C18—C19—N1	−0.2 (5)
O4—Cd1—O2—C1	141.5 (2)	C25—C18—C19—N1	179.5 (3)
O1—Cd1—O3—C5	85.7 (3)	C17—C18—C19—C20	179.0 (3)
N2—Cd1—O3—C5	−105.85 (18)	C25—C18—C19—C20	−1.3 (5)
N1—Cd1—O3—C5	−176.28 (18)	C24—N2—C20—C21	−1.6 (5)
O7—Cd1—O3—C5	94.65 (18)	Cd1—N2—C20—C21	175.1 (2)
O4—Cd1—O3—C5	−2.24 (16)	C24—N2—C20—C19	177.4 (3)
O2—Cd1—O3—C5	−29.5 (2)	Cd1—N2—C20—C19	−5.9 (3)
O1—Cd1—O4—C5	−156.55 (18)	N1—C19—C20—N2	0.1 (4)
O3—Cd1—O4—C5	2.24 (16)	C18—C19—C20—N2	−179.1 (3)
N2—Cd1—O4—C5	86.65 (18)	N1—C19—C20—C21	179.2 (3)
N1—Cd1—O4—C5	12.2 (2)	C18—C19—C20—C21	−0.1 (4)
O7—Cd1—O4—C5	−83.06 (18)	N2—C20—C21—C22	1.2 (5)
O2—Cd1—O4—C5	163.94 (19)	C19—C20—C21—C22	−177.8 (3)
Cd1—O2—C1—O1	−7.0 (3)	N2—C20—C21—C26	−179.9 (3)
Cd1—O2—C1—C2	169.4 (3)	C19—C20—C21—C26	1.1 (5)
Cd1—O1—C1—O2	8.0 (4)	C20—C21—C22—C23	−0.1 (6)
Cd1—O1—C1—C2	−168.4 (2)	C26—C21—C22—C23	−179.0 (4)
O2—C1—C2—C4	−16.4 (5)	C21—C22—C23—C24	−0.5 (7)
O1—C1—C2—C4	160.2 (3)	C20—N2—C24—C23	0.9 (6)
O2—C1—C2—C3	165.2 (3)	Cd1—N2—C24—C23	−175.4 (3)
O1—C1—C2—C3	−18.3 (4)	C22—C23—C24—N2	0.1 (7)
C4—C2—C3—C4^i^	0.5 (5)	C17—C18—C25—C26	−178.6 (4)
C1—C2—C3—C4^i^	179.0 (3)	C19—C18—C25—C26	1.7 (6)
C3—C2—C4—C3^i^	−0.5 (5)	C18—C25—C26—C21	−0.6 (6)
C1—C2—C4—C3^i^	−179.0 (3)	C22—C21—C26—C25	178.0 (4)
Cd1—O4—C5—O3	−4.0 (3)	C20—C21—C26—C25	−0.8 (6)

Symmetry codes: (i) −*x*+1/2, −*y*+3/2, −*z*+1; (ii) −*x*+1/2, −*y*+3/2, −*z*; (iii) −*x*, *y*, −*z*+1/2.

### Hydrogen-bond geometry (Å, °)

**Table:**

*D*—H···*A*	*D*—H	H···*A*	*D*···*A*	*D*—H···*A*
O5—H5···O6^iv^	0.82	1.92	2.728 (3)	167
O7—H7A···O2^v^	0.85	1.88	2.665 (3)	154
O7—H7B···O4^v^	0.85	2.28	3.019 (3)	146

Symmetry codes: (iv) *x*, *y*+1, *z*; (v) −*x*+1/2, *y*+1/2, −*z*+1/2.
